# Validation of colorectal cancer surgery data from administrative data sources

**DOI:** 10.1186/1471-2288-12-97

**Published:** 2012-07-11

**Authors:** Xue Li, Charlotte King, Christopher deGara, Jonathon White, Marcy Winget

**Affiliations:** 1 Division of Community Oncology, Cancer Care, Alberta Health Services, 1500-10123 99 Street, T5J 3H1, Edmonton, Alberta, Canada; 2 Division of Continuous Professional Learning, Faculty of Medicine and Dentistry, University of Alberta, 2-590 Edmonton Clinic Health Academy, 11405-87th Avenue NW, T6G 1C9, Edmonton, Alberta, Canada; 3 Department of Surgery, Faculty of Medicine and Dentistry, University of Alberta, 2D, Walter C Mackenzie Health Sciences Centre, 8440 - 112 Street, T6G 2B7, Edmonton, Alberta, Canada; 4 Department of Public Health Sciences, School of Public Health, University of Alberta, 3-300 Edmonton Clinic Health Academy, 11405-87th Avenue NW, T6G 1C9, Edmonton, Alberta, Canada; 5 1500 Sun Life Place, 10123 99 Street NW, Edmonton, Alberta, T5J 3H1, Canada

**Keywords:** Colorectal cancer, Surgery, Data validation, Administrative data, Sensitivity, Specificity, Positive predictive value, Negative predictive value

## Abstract

**Background:**

Surgery is the primary treatment for colorectal cancer for both curative and palliative intent. Availability of high quality surgery data is essential for assessing many aspects of the quality of colorectal cancer care. The objective of this study was to determine the quality of different administrative data sources in identifying surgery for colorectal cancer with respect to completeness and accuracy.

**Methods:**

All residents in Alberta, Canada who were diagnosed with invasive colorectal cancer in years 2000-2005 were identified from the Alberta Cancer Registry and included in the study. Surgery data for these patients were obtained from the Cancer Registry (which collects the date of surgery for which the primary tumor was removed) and compared to surgery data obtained from two different administrative data sources: Physician Billing and Hospital Inpatient data. Sensitivity, specificity, positive predictive value, negative predictive value and observed agreement were calculated compared to the Cancer Registry data.

**Results:**

The Physician Billing data alone or combined with Hospital Inpatient data demonstrated equally high sensitivity (97% for both) and observed agreement with the Cancer Registry data (93% for both) for identifying surgeries. The Hospital Inpatient data, however, had the highest specificity (80%). The positive predictive value varied by disease stage and across data sources for stage IV (99% for stages I-III and 83-89% for stage IV), the specificity is better for colon cancer surgeries (72-85%) than for rectal cancer surgeries (60-73%); validation measures did not vary over time.

**Conclusion:**

Physician Billing data identify the colorectal cancer surgery more completely than Hospital Inpatient data although both sources have a high level of completeness.

## Background

Surgical resection is the primary treatment for colorectal cancer, resulting in cure in 80% of patients [1-4]. Even if cure is not possible, palliative surgery may be needed to control symptoms such as pain, bleeding, obstruction or perforation [3,4]. Maintaining high quality surgery data is, therefore, essential to measuring many aspects of the quality of colorectal cancer care including adherence to treatment guidelines and evaluation of patient treatment outcomes.

A well-established cancer registry in many countries provides the primary data source used for identifying cancer diagnosis and sometimes treatment. When cancer registries do collect treatment information, it has been shown to have high quality [5] and such registries are central in enabling assessment of the quality of cancer care [6,7]. Each registry, however, is managed to meet the expectations for its primary role [7]. In many instances, the primary role of a cancer registry is only to monitor cancer incidence and death for surveillance purposes. Treatment data are, therefore, not routinely collected by all cancer registries, nor, when collected, are they collected in the same way. For instance, some cancer registries do not collect any treatment information, others collect only the surgery to remove the primary tumor and others collect all surgeries related to the cancer.

Variation in data collection patterns of cancer treatment by cancer registries makes it challenging to conduct comparison studies across jurisdictions related to treatment patterns, adherence to treatment guidelines, or similar studies. Such studies are important for understanding variation in cancer survival and cancer prevalence across jurisdictions and may help inform ideas for implementing changes in cancer care service delivery.

In the absence of standardized collection and coding of treatment data by a cancer registry or other data source, administrative data are the most available data sources for basic information related to receipt of treatment, particularly hospital-based treatment [8,9]. Because administrative data are not developed for research or quality assessment purposes, it is imperative to be aware of the strengths and weaknesses of the data prior to using them in order to avoid making and disseminating misleading or inaccurate information [10]. Data validation is a critical step towards understanding potential biases that may be created by using such data. The purpose of this study, therefore, was to evaluate the completeness of different administrative data sources in identifying surgery for colorectal cancer compared to a cancer registry that collects the date of surgery conducted for removal of the primary tumor.

## Methods

### Inclusion and exclusion criteria

All residents of Alberta, Canada who were diagnosed with invasive colon (International Classification of Diseases for Oncology (ICD-O) [11] codes: c18.0, c18.2-c18.9) or rectal (ICD-O c19.9 and c20.9) cancer in 2000-2005 were identified from the Alberta Cancer Registry and included in the study. Patients were excluded if they had stage 0 cancer, a histology that is not staged according to the Collaborative Staging Guidelines for colorectal cancer (for example, sarcomas) [12], or missing the unique life time identifier (ULI) needed to link the data across the multiple data sources. The ULI is a code that uniquely identifies individuals enrolled in the Alberta Health Care Insurance Plan, the universal healthcare insurance provider for all residents of Alberta, Canada. Once assigned, an individual’s code does not change even if s/he moves in and out of the province. Patients for whom their disease stage was missing for reasons other than having a histology that could not be staged were included.

### Data from the Alberta cancer registry

Diagnosis and surgery dates were obtained from the Alberta Cancer Registry. In addition to its legislative mandate to register and code all cancer diagnoses in the province, the Alberta Cancer Registry also collects and maintains demographic and clinical information, including all treatment modalities for the initial diagnosis and start dates of each modality. The date of surgery recorded in the cancer registry is the date of removal of the primary tumor based on pathology and surgical reports and does not include surgery to metastatic sites; only one surgery date per cancer diagnosis is recorded. All labs, hospitals and clinicians are required by law to report all cancer cases and furnish any additional information requested by the Alberta Cancer Registry. The Alberta Cancer Registry is reviewed annually by the North American Association of Comprehensive Cancer Registries (NAACCR) to ascertain the quality and completeness of its data and is regularly awarded the highest level of certification [13].

### Administrative data sources

Colorectal surgery data were obtained from two provincial administrative databases: 1) the Discharge Abstract Database (Hospital Inpatient data) which records diagnosis and procedures on all admissions to hospitals in Alberta; and 2) the Physician Billing database, which contains all billing claims submitted by physicians remunerated on a fee-for-service basis. From each of these databases, dates and codes for the first colorectal surgery were identified and included that occurred 7 days prior to or up to 548 days (1.5 years) after the diagnosis date. The lower bound of the window was to address potential inaccuracy of dates in the Physician Billing data and the upper bound was based on the maximum time from diagnosis to surgery that was observed in the Cancer Registry surgery dates. The date of the first colorectal cancer surgery within the time window was identified from each data source and included in the study. In practice, if more than one surgery is conducted related to colorectal cancer, the first is expected to be for the removal of the primary tumor as subsequent surgeries are generally for rectal reconstruction, stoma removal, or similar; thus the first surgery is expected to be the same as the surgery recorded in the Cancer Registry.

The Physician Billing and Hospital Inpatient data were selected for the study because: 1) almost all surgeons are paid fee-for-service, therefore Physician Billing should capture surgeries well and 2) colorectal surgery can only be conducted on an inpatient basis, therefore, the Hospital Inpatient database should also be fairly complete. Additionally, trained and certified Health Records Technicians are responsible for coding diagnoses and procedures that are entered into the Hospital Inpatient database so information should be accurate.

The time period was chosen because there was a change in the coding schema used for coding procedures in the Hospital Inpatient data in April 2002 and we wanted to assess whether these changes would impact the data validity. Physician billing uses Canadian Classification of Procedures (CCP) coding system; Hospital Inpatient prior to April 2002 used the ICD-9-Clinical Modification (CM) coding system and switched to Canadian Classification of Health Interventions (CCI) coding system in April 2002. Colorectal surgery codes were identified for each data source and coding system with input from local physicians and a literature search to ensure all appropriate codes were included. The complete list of the colorectal surgery codes included is in Additional file 1.

A dataset was also created that combined the two administrative datasets to determine if combining surgery information enhanced the completeness and validity of the data over either of the single administrative data sources. Data were combined using the following rules: 1) if a surgery date for a patient was only in one of the data sources then that date was included in the combined dataset; 2) if the data sources had two different surgery dates, the earlier date was included in the combined dataset.

The databases used in the study are not publicly available. The Alberta Cancer Registry data were made available upon ethics approval. The provincial administrative databases are governed by the provincial ministry, Alberta Health and Wellness (AHW). AHW provided the provincial administrative data required for the study after conducting a Privacy Impact Assessment and signing a confidentiality agreement. Ethics approval for the study was obtained from the Alberta Cancer Research Ethics Board.

### Data analysis

The date of surgery in the Alberta Cancer Registry was considered to be the source of truth. Sensitivity, specificity, positive predictive value (PPV) and negative predictive value (NPV) were calculated for the Physician Billing data, the Hospital Inpatient data, and the combined administrative dataset. The above measures were calculated overall and by year of diagnosis, stage at diagnosis, and tumor site; these factors were chosen because they were considered to be the factors by which there would most likely be variation in completeness of the administrative data. The observed agreement was calculated to test the strength of agreement instead of the Kappa statistic because the latter is influenced substantially by trait prevalence and imbalanced marginal distributions [14-18]. In our context, this is particularly relevant in the analysis by stage as more than 98% of patients with stages I-III disease received surgery. Although there are not set standards for defining excellent, good, acceptable, and poor related to observed agreement, it is reasonable to define “excellent” as 90-100% and “good” as 80-89%. The 95% confidence interval for each estimate was calculated. Unstable estimates, defined as having a 95% confidence interval wider than 15% or a width that was 40% or more than the estimate, are noted in the tables. In order to allow ease of comparison of validation measures across datasets in the main tables, confidence intervals are presented in Additional file 2: Appendices B-D only. All analyses were performed using statistical software SAS 9.1.3 (SAS Institute, Cary, NC, USA) or STATA/SE 10.0 (StataCorp LP, TX, USA).

## Results

A total of 8,533 patients residing in Alberta were diagnosed with invasive colorectal cancer in years 2000-2005. There were 225 patients excluded from the study for the following reasons: 2 were missing their ULI; 140 had a cancer histology that cannot be staged using Collaborative Staging rules for colorectal cancer; and 83 patients had a stage 0 cancer. The remaining 8,308 colorectal cancer patients were included in the study.

Table 1 compares the number and percentage of patients who had surgery according to each of the Alberta Cancer Registry, Physician Billing, Hospital Inpatient data, and the combined administrative dataset. There were 7,066 (85%) patients who had surgery according to the Alberta Cancer Registry out of 8,308 colorectal cancer patients diagnosed in years 2000-2005. Both administrative datasets alone or combined identified similar numbers and percentages of patients who had surgery as the Cancer Registry for all years combined and by year of diagnosis, although the number identified in the Physician Billing was slightly higher than other data sources in all diagnosis years. The greatest differences between data sources were for patients diagnosed with stage I disease and with stage IV disease. In the case of stage I patients, the cancer registry recorded more surgeries than the administrative data sources (99% of patients vs. 87-91%, respectively). Conversely, the Cancer Registry identified fewer patients who had surgery with stage IV disease than the administrative datasets (60% vs. 66-74%, respectively).

**Table 1:** Colorectal surgery patients identified by data source of cancer registry, physician billing and hospital inpatient data

		**Surgery Patients Identified from Each Data Source**							
**Factors**	**All Patients**	**Cancer Registry**		**Physician Billing**		**Hospital Inpatient**		**Combined Administrative**	
	**n**	**n**	**(%)**^**1**^	**n**	**(%)**^**1**^	**n**	**(%)**^**1**^	**n**	**(%)**^**1**^
	8308	7066	(85)	7173	(86)	6905	(83)	7241	(87)
**Year**									
2000	1324	1149	(87)	1168	(88)	1131	(85)	1183	(89)
2001	1331	1126	(85)	1155	(87)	1109	(83)	1166	(88)
2002	1337	1152	(86)	1170	(88)	1135	(85)	1181	(88)
2003	1432	1231	(86)	1232	(86)	1200	(84)	1250	(87)
2004	1418	1176	(83)	1202	(85)	1152	(81)	1210	(85)
2005	1466	1232	(84)	1246	(85)	1194	(81)	1257	(86)
**Stage**									
I	1387	1379	(99)	1251	(90)	1204	(87)	1259	(91)
II	2207	2166	(98)	2167	(98)	2125	(96)	2174	(99)
III	1902	1875	(99)	1867	(98)	1835	(96)	1870	(98)
IV	1953	1164	(60)	1396	(71)	1293	(66)	1436	(74)
Missing	859	482	(56)	492	(57)	464	(54)	508	(59)
**Tumor Site**									
Colon	5303	4534	(85)	4585	(86)	4429	(84)	4608	(87)
Rectum	3005	2532	(84)	2588	(86)	2492	(83)	2639	(88)

^1^ Percent is based on “All Patients” in each row.

Table 2 summarizes the validation measures for the administrative data sources compared to the Cancer Registry overall and by year, stage and tumor site. The validation measures and their corresponding confidence intervals are shown in Additional file 1. The Physician Billing data alone and combined with Hospital Inpatient data have similar high sensitivity (97%) and PPV (95%). Conversely, the Hospital Inpatient data has higher specificity than the Physician Billing, 80% vs. 72%, respectively. The observed agreement with the cancer registry is over 90% for all the administrative datasets except for patients diagnosed with stage IV or unknown stage disease, where it is 84-89% depending on the stage and dataset. Similarly, the PPV is 99% for disease stages I-III in all datasets but lower for patients diagnosed at stage IV or unknown stage (range 80-89%). Estimates for specificity and NPV are unstable for stage I-III patients in all data sources, because of the very small number of patients who did not have surgery in all the data sources. There is a common trend across the data sources that all measures for rectal cancer are lower than for colon cancer.

**Table 2:** **Validation measures**^**1**^**for colorectal surgery in physician billing and hospital inpatient data compared to the Alberta Cancer Registry overall and by year of diagnosis, stage and tumor site**

	**Physician Billing Data**					**Hospital Inpatient Data**					**Combined Administrative Data**				
					**Observed**					**Observed**					**Observed**
**Factors**	**Sens.**	**Spec.**	**PPV**	**NPV**	**Agreement**	**Sens.**	**Spec.**	**PPV**	**NPV**	**Agreement**	**Sens.**	**Spec.**	**PPV**	**NPV**	**Agreement**
	%	%	%	%	%	%	%	%	%	%	%	%	%	%	%
**All**	97	72	95	79	93	94	80	96	72	92	97	68	95	79	93
**Year**															
2000	97	69^2^	95	77^2^	93	96	83^2^	97	75^2^	94	97	64^2^	95	79^2^	93
2001	97	67^2^	94	78^2^	92	95	80^2^	96	74^2^	93	97	62^2^	93	77^2^	91
2002	97	70^2^	95	77^2^	93	95	77^2^	96	70^2^	92	97	65^2^	95	78^2^	92
2003	97	80^2^	97	80^2^	94	94	81^2^	97	70^2^	92	97	74^2^	96	81^2^	94
2004	97	74^2^	95	83^2^	93	94	83^2^	96	76^2^	92	97	72^2^	94	84^2^	93
2005	96	74^2^	95	78^2^	92	93	79^2^	96	68^2^	91	96	69^2^	94	77^2^	92
**Stage**															
I	91	88^3^	100	5^3^	91	87	88^3^	100	4^3^	87	91	88^3^	100	5^3^	91
II	99	49^3^	99	50^3^	98	97	63^3^	99	32^3^	97	99	41^3^	99	52^3^	98
III	99	70^3^	99	54^3^	99	98	78^3^	100	31^3^	97	99	70^3^	100	59^3^	99
IV	99	69^2^	82	98	87	97	79	87	94	89	99	64^2^	80	98	85
Missing	88	82^2^	86^2^	84^2^	85	85^2^	86^2^	89	82^2^	85	88	78^2^	84^2^	84^2^	84
**Tumor Site**															
Colon	97	75^2^	96	80	94	95	85	97	75	94	97	73^2^	95	80	93
Rectal	96	69^2^	94	78^2^	92	93	73^2^	95	67^2^	90	97	60^2^	93	78^2^	91

^1^ The width of the 95% confidence interval for estimates without a superscript are < 6%.

^2^ Width of the 95% confidence interval is 6 – 14%.

^3^ Unstable estimate: width of 95% confidence interval is >14% and/or the width of the interval is >40% of the value of the estimate.

To examine the accuracy of surgery dates identified from administrative data sources, the date of surgery based on the Cancer Registry was subtracted from the date of surgery based on each of the administrative datasets. Over 90% of the surgery dates in both the individual and combined administrative datasets matched the dates in the Cancer Registry exactly (not shown in the tables). This confirms that the accuracy of dates in all the data sources, even Physician Billing, is quite good. It also confirms the assumption that the first surgery in any of the administrative data sources corresponds to the surgery recorded in the Cancer Registry, that is, removal of the primary tumor, was correct.

## Discussion

The aim of the study was to assess two different administrative data sources with respect to completeness and accuracy of colorectal cancer surgery data compared to a cancer registry that collects only the date of the surgery responsible for removal of the primary tumor. The findings of the study support the validity and comparability of colorectal surgery data from administrative data sources for this purpose. Specifically, Physician Billing data alone or combined with Hospital Inpatient data, are the most comparable alternative data sources to a cancer registry for identifying the date of the surgery in which the primary colorectal tumor was removed.

The largest discrepancy between the cancer registry surgery data and the administrative data sources occurred for patients with stage I and stage IV disease. Specifically, proportionally more surgeries for stage I cancer patients were identified in the cancer registry than either of the administrative data sources and, conversely, fewer were found in the cancer registry for stage IV patients than in the administrative data sources; this is likely due to rules used by the Alberta Cancer Registry in defining surgery. The cancer registry defines surgery as the event that results in excision of the primary tumor. A polypectomy in a stage I patient, therefore, may be coded as a surgery in the Alberta Cancer Registry; this will be the case if no further excision is required to ensure negative margins. It may be possible to create an algorithm using administrative data that mimics more closely the rules of the cancer registry for stage I patients who receive polypectomy rather than an inpatient surgery; the current study, however, was focused on the comparability of the data sources specifically using surgery codes from the administrative data sources.

With respect to stage IV patients, again, the Alberta Cancer Registry only captures the surgery responsible for removal of the primary tumor. Some surgeries on patients with stage IV colorectal cancer are de-bulking as opposed to removing the majority of the tumor. Some may be done only to create a stoma or re-route the colon. It is expected that all of these surgeries will be identified in the administrative data sources but not in a registry that captures only removal of the primary tumor. A clear interpretation of results must be based on fully understanding of coding rules and limitation of each data source.

In addition to disease stage, consistency of the validation measures over time was evaluated. None of the validation measures varied over time for any of the data sources indicating the reliability of the administrative data sources, even over a time period in which coding systems changed. Changes made to reimbursement policies for colorectal surgeries in future years, however, could impact the generalizability of this conclusion, so need to be considered. The fact that there were not changes in the values of the validation measures for the Hospital Inpatient data, even though there was a change in coding from ICD-9-CM to CCI codes over the study period, is a reflection of the robustness of the coding systems for colorectal surgery and also of the quality and consistency of the training received by health records technicians responsible for coding the hospital data.

The results of our study suggest that individual Physician Billing data can identify 1% more surgeries or 2% more if combined with Hospital Inpatient data compared to the surgery records identified from Cancer Registry. Depending on the circumstances, this small difference may or may not matter for addressing a research or quality question. The high level of completeness and accuracy in Physician Billing data alone and in combination with Hospital Inpatient data indicates that administrative data can serve as excellent sources to identify cancer surgeries. The findings are consistent with other studies conducted in North America that assessed the completeness and validity of administrative data sources for identifying breast cancer surgery [8,19-21]. The findings of this study are likely generalizable to other jurisdictions which have universal health insurance and/or for which surgeons are remunerated primarily on a fee-for-service basis and for similar procedures that are performed primarily or only in a hospital.

In addition to cross-jurisdiction comparisons, another potentially important application of administrative data as the source for cancer surgery data compared to a data source such as a cancer registry, is the relative quick availability of the data. It takes the Alberta Cancer Registry 18 months to 2 years to complete the annual coding of all cancer cases including treatment information; this time period is probably typical of other registries that belong to NAACCR given the organization’s reporting period requirements. Hospital Inpatient data and physician billing data, however, are generally available in much closer proximity to the date a treatment such as surgery occurs, approximately three to six months. Physician billing and Hospital Inpatient data can, therefore, be used for monitoring high level quality measures, measuring the impact of certain types of changes to the health care system, and facilitating informed responsiveness to issues within the health care system as they arise. In spite of these strengths, an important limitation, however is that administrative data by themselves cannot be used to address questions that require information related to clinical case mix of patients.

## Conclusions

In conclusion, both Physician Billing and Hospital Inpatient data are valid sources for identifying the date of the surgery responsible for removing the primary colorectal cancer. Accuracy of the physician billing data, however, is subject to the fee code policy. Caution is needed in the conduct and interpretation of studies based on physician billing data; strong understanding of the way in which physicians use billing codes and the percentage of physicians who perform the procedure of interest that bill for it is needed. Validation of the data is also critical.

## Competing interests

The authors declare that they have no competing interests.

## Authors’ contributions

MW oversaw the study, obtained financial support, and finalized the manuscript; MW and CK designed the study; CK and XL analyzed the data; CG and JW provided clinical expertise and provided input on manuscript drafts; CK, XL and MW drafted the manuscript; all authors gave approval of the final manuscript.

## Pre-publication history

The pre-publication history for this paper can be accessed here:

http://www.biomedcentral.com/1471-2288/12/97/prepub

## Supplementary Material

Additional file 1: Appendix A. Colorectal Surgery Codes. Click here for file

Additional file 2: Appendices B-D. Tables with 95% confidence intervals. Click here for file

## References

[B1] Mirza MS Longman RJ Farrokhyar F Sheffield JP Kennedy RH Long-term outcomes for laparoscopic versus open resection of nonmetastatic colorectal cancer J Laparoendosc Adv Surg Tech A 2008 18 679 685 10.1089/lap.2007.0169 18699750

[B2] Kahnamoui K Cadeddu M Farrokhyar F Anvari M Laparoscopic surgery for colon cancer: a systematic review Can J Surg 2007 50 48 57 17391617 PMC2384248

[B3] Nenshi R Baxter N Kennedy E Schultz SE Gunraj N Wilton AS Urbach DR Simunovic M Urbach DR, Simunovic M, Schultz SE Surgery for Colorectal Cancer Cancer Surgery in Ontario: ICES Atlas 2008 Toronto, ON: Institute for Clinical Evaluative Sciences

[B4] Lavery IC Lopez-Kostner F Pelley RJ Fine RM Treatment of colon and rectal cancer Surg Clin North Am 2000 80 535 569 ix 10.1016/S0039-6109(05)70200-0 10836006

[B5] Turner D Hildebrand KJ Fradette K Latosinsky S Same question, different data source, different answers? Data source agreement for surgical procedures on women with breast cancer Healthc Policy 2007 3 46 54 19305755 PMC2645122

[B6] Lipscomb J Gillespie TW State-level cancer quality assessment and research: building and sustaining the data infrastructure Cancer J 2011 17 246 256 10.1097/PPO.0b013e3182296422 21799333 PMC4879873

[B7] Beatty JD Adachi M Bonham C Atwood M Potts MS Hafterson JL Aye RW Utilization of cancer registry data for monitoring quality of care Am J Surg 2011 201 645 649 10.1016/j.amjsurg.2011.01.004 21545915

[B8] Du X Freeman JL Warren JL Nattinger AB Zhang D Goodwin JS Accuracy and completeness of Medicare claims data for surgical treatment of breast cancer Med Care 2000 38 719 727 10.1097/00005650-200007000-00004 10901355

[B9] Meguerditchian AN Stewart A Roistacher J Watroba N Cropp M Edge SB Claims data linked to hospital registry data enhance evaluation of the quality of care of breast cancer J Surg Oncol 2010 101 593 599 10.1002/jso.21528 20461766

[B10] Abraham NS Cohen DC Rivers B Richardson P Validation of administrative data used for the diagnosis of upper gastrointestinal events following nonsteroidal anti-inflammatory drug prescription Aliment Pharmacol Ther 2006 24 299 306 10.1111/j.1365-2036.2006.02985.x 16842456

[B11] Fritz A, Percy C, Jack A, Shanmugaratnam K, Sobin L, Parkin DM, Whelan S International Classification of Diseases for Oncology 2000 Geneva, Switzerland: World Health Organization

[B12] Collaborative Staging Task Force of the American Joint Committee on Cancer Collaborative Staging Manual and Coding Instructions, version 01.04.00. NIH Publication Number 04-5496. Incorporates updates through September 8, 2006 2004 Jointly published by American Joint Committee on Cancer (Chicago, IL) and U.S. Department of Health and Human Services (Bethesda, MD), Chicago, IL

[B13] Tucker TC Howe HL Weir HK Certification for population-based cancer registries J Reg Mgmt 1999 26 24 27

[B14] Gwet K Inter-rater reliability: dependency on trait prevalence and marginal homogeneity 2002 Volume 2nd

[B15] Viera AJ Garrett JM Understanding interobserver agreement: the kappa statistic Fam Med 2005 37 360 363 15883903

[B16] Soeken KL Prescott PA Issues in the use of kappa to estimate reliability Med Care 1986 24 733 741 10.1097/00005650-198608000-00008 3736144

[B17] Feinstein AR Cicchetti DV High agreement but low kappa: I. The problems of two paradoxes J Clin Epidemiol 1990 43 543 549 10.1016/0895-4356(90)90158-L 2348207

[B18] Cicchetti DV Feinstein AR High agreement but low kappa: II resolving the paradoxes J Clin Epidemiol 1990 43 551 558 10.1016/0895-4356(90)90159-M 2189948

[B19] Pinfold SP Goel V Sawka C Quality of hospital discharge and physician data for type of breast cancer surgery Med Care 2000 38 99 107 10.1097/00005650-200001000-00011 10630724

[B20] Kahn LH Blustein J Arons RR Yee R Shea S The validity of hospital administrative data in monitoring variations in breast cancer surgery Am J Public Health 1996 86 243 245 10.2105/AJPH.86.2.243 8633744 PMC1380336

[B21] Cooper GS Virnig B Klabunde CN Schussler N Freeman J Warren JL Use of SEER-Medicare data for measuring cancer surgery Med Care 2002 40 IV-8 10.1097/00005650-200208001-00006 12187167

