# Supplementary material for: Validation of colorectal cancer surgery data from administrative data sources
Source: BMC Med Res Methodol. 2012 Jul 11;12:97. doi: 10.1186/1471-2288-12-97 (PMC3406984; doi:10.1186/1471-2288-12-97)
Supplement: Additional file 1: — Appendix A. Colorectal Surgery Codes. [file 1471-2288-12-97-S1.doc]

**Appendix A: Colorectal Surgery Codes**

The Canadian Classification of Procedures (CCP) – physician billing, all years

| 57.53 | Right hemicolectomy |
| --- | --- |
| 57.6 A | Total colectomy {Total colectomy with or without ileostomy} |
| 57.6 B | Total proctocolectomy with ileostomy |
| 57.6 C | Total proctocolectomy with continent ileostomy |
| 57.6 D | Total proctocolectomy with diverting ileostomy, ileo-anal pouch and ileo-anal anastomosis |
| 57.6 E | Creation of ileo-anal pouch and ileo-anal anastomosis following previous total colectomy |
| 57.55 | Left hemicolectomy |
| 57.59A | Other partial excision of large intestine {Segmental colectomy} |
| 58.44A | Other revision of stoma of large intestine {Colostomy revision} |
| 58.53A | Closure of stoma of large intestine {Colostomy closure} |
| 58.81A | Intra-abdominal manipulation of intestine, unqualified {Any form of obstruction without resection} |
| 58.81B | Intra-abdominal manipulation of intestine, unqualified {Any form of obstruction with enterotomy decompression} |
| 58.81C | Intra-abdominal manipulation of intestine, unqualified {Any form of obstruction with resection} |
| 60.4A | Abdominal-perineal resection |
| 60.4B | Abdominal-perineal resection of rectum {Abdominal-perineal resection} |
| 60.52A | Other anterior resection {Anterior segmental resection, rectosigmoid} |
| 60.59A | Other resection of rectum NEC {Perineal resection of rectum} |
| 60.59B | Other resection of rectum NEC {Trans-sphincteric resection of rectum} |

International Statistical Classification of Diseases, Injuries, and Causes of Death, Ninth Revision -Clinical Modification (ICD-9-CM) – Inpatient Hospital data, 1994 to March 2002

| 4573 | Right hemicolectomy |
| --- | --- |
| 4574 | Resection of transverse colon |
| 4575 | Left hemicolectomy |
| 4576 | Sigmoidectomy |
| 4579 | Other part excision large intestine |
| 458 | Total intra-abdominal colectomy |
| 4593 | Other small-to-large intestinal anastomosis |
| 4594 | Large-to-large intestinal anastomosis |
| 4595 | Intestinal anastomosis to anus |
| 4610 | Colostomy not otherwise specified |
| 4643 | Other revision stoma large intestine |
| 4652 | Closure stoma large intestine |
| 4681 | Intra-abdominal manipulation small intestine |
| 4682 | Intra-abdominal manipulation large intestine |
| 4849 | Other pull-through resection rectum |
| 485 | Abdominoperineal resection rectum |
| 4862 | Anterior resection rectum with colostomy |
| 4863 | Other anterior resection rectum |
| 4869 | Other resection of rectum |
| 4879 | Other repair of rectum |
| 543 | Excision or destruction of lesion or tissue of abdominal wall |

| The Canadian Classification of Health Interventions (CCI) – Inpatient Hospital data, April 2002 to present: | |
| --- | --- |
| 1NM76 | colon bypass |
| 1NM77 | colon bypass with exteriorization |
| 1NM87 | colon excision partial |
| 1NM91 | colon excision radical |
| 1NM89 | colon excision total |
| 1NQ87 | rectum excision partial |
| 1NQ89 | rectum excision total |

All codes were selected based solely on the first three fields (5 characters).
