# Supplementary material for: Validation of colorectal cancer surgery data from administrative data sources
Source: BMC Med Res Methodol. 2012 Jul 11;12:97. doi: 10.1186/1471-2288-12-97 (PMC3406984; doi:10.1186/1471-2288-12-97)
Supplement: Additional file 2: — Appendices B-D. Tables with 95% confidence intervals. [file 1471-2288-12-97-S2.doc]

**Appendix B**

Validation measures and 95% confidence intervals for colorectal surgery in physician billing data compared to the Alberta Cancer Registry overall and by year of diagnosis, stage and tumor site

|  | **Physician Billing Data** | | | | | | | | | |
| --- | --- | --- | --- | --- | --- | --- | --- | --- | --- | --- |
| **Factors** | **Sensitivity** | | **Specificity** | | **PPV** | | **NPV** | | **Observed Agreement** | |
|  | % | (95% CI) | % | (95% CI) | % | (95% CI) | % | (95% CI) | % | (95% CI) |
| **All** | 97 | (96-97) | 72 | (70-75) | 95 | (95-96) | 79 | (77-81) | 93 | (92 -94) |
| **Year** |  |  |  |  |  |  |  |  |  |  |
| 2000 | 97 | (96-98) | 69 | (61-75) | 95 | (94-96) | 77 | (70-83) | 93 | (92-94) |
| 2001 | 97 | (95-98) | 67 | (60-74) | 94 | (93-95) | 78 | (72-84) | 92 | (91-93) |
| 2002 | 97 | (96-98) | 70 | (63-76) | 95 | (94-96) | 77 | (70-83) | 93 | (92-94) |
| 2003 | 97 | (96-98) | 80 | (73-85) | 97 | (96-98) | 80 | (74-85) | 94 | (93-95) |
| 2004 | 97 | (96-98) | 74 | (68-79) | 95 | (93-96) | 83 | (77-88) | 93 | (92-94) |
| 2005 | 96 | (95-97) | 74 | (67-79) | 95 | (94-96) | 78 | (72-83) | 92 | (91-93) |
| **Stage** |  |  |  |  |  |  |  |  |  |  |
| I | 91 | (89-92) | 88 | (47-100) | 100 | (99-100) | 5 | (2-10) | 91 | (89-93) |
| II | 99 | (98-99) | 49 | (33-65) | 99 | (98-99) | 50 | (34-66) | 98 | (97-99) |
| III | 99 | (99-100) | 70 | (50-86) | 99 | (99-100) | 54 | (37-71) | 99 | (99-99) |
| IV | 99 | (98-99) | 69 | (66-72) | 82 | (80-84) | 98 | (96-99) | 87 | (86-88) |
| Missing | 88 | (85-91) | 82 | (77-85) | 86 | (83-89) | 84 | (80-88) | 85 | (83-87) |
| **Tumor Site** |  |  |  |  |  |  |  |  |  |  |
| Colon | 97 | (96-97) | 75 | (71-78) | 96 | (95-96) | 80 | (77-83) | 94 | (93-95) |
| Rectal | 96 | (96-97) | 69 | (64-73) | 94 | (93-95) | 78 | (74-82) | 92 | (91-93) |

**Appendix C**

Validation measures and 95% confidence intervals for colorectal surgery in hospital inpatient data compared to the Alberta Cancer Registry overall and by year of diagnosis, stage and tumor site.

|  | **Hospital Inpatient Data** | | | | | | | | | |
| --- | --- | --- | --- | --- | --- | --- | --- | --- | --- | --- |
| **Factors** | **Sensitivity** | | **Specificity** | | **PPV** | | **NPV** | | **Observed Agreement** | |
|  | % | (95% CI) | % | (95% CI) | % | (95% CI) | % | (95% CI) | % | (95% CI) |
| **All** | 94 | (94-95) | 80 | (78-83) | 96 | (96-97) | 72 | (70-74) | 92 | (91-93) |
| **Year** |  |  |  |  |  |  |  |  |  |  |
| 2000 | 96 | (95-97) | 83 | (76-88) | 97 | (96-98) | 75 | (68-81) | 94 | (93-95) |
| 2001 | 95 | (93-96) | 80 | (74-85) | 96 | (95-97) | 74 | (68-80) | 93 | (92-94) |
| 2002 | 95 | (93-96) | 77 | (70-83) | 96 | (95-97) | 70 | (63-77) | 92 | (91-93) |
| 2003 | 94 | (93-96) | 81 | (74-86) | 97 | (96-98) | 70 | (63-76) | 92 | (91-93) |
| 2004 | 94 | (93-96) | 83 | (78-88) | 96 | (95-97) | 76 | (70-81) | 92 | (91-93) |
| 2005 | 93 | (91-94) | 79 | (73-84) | 96 | (95-97) | 68 | (62-74) | 91 | (90-92) |
| **Stage** |  |  |  |  |  |  |  |  |  |  |
| I | 87 | (85-89) | 88 | (47-100) | 100 | (99-100) | 4 | (2-8) | 87 | (85-89) |
| II | 97 | (97-98) | 63 | (47-78) | 99 | (99-100) | 32 | (22-43) | 97 | (96-98) |
| III | 98 | (97-98) | 78 | (58-91) | 100 | (99-100) | 31 | (21-44) | 97 | (96-98) |
| IV | 97 | (95-98) | 79 | (76-82) | 87 | (85-89) | 94 | (92-96) | 89 | (88-90) |
| Missing | 85 | (82-88) | 86 | (82-89) | 89 | (85-91) | 82 | (78-86) | 85 | (83-87) |
| **Tumor Site** |  |  |  |  |  |  |  |  |  |  |
| Colon | 95 | (94-96) | 85 | (82-87) | 97 | (97-98) | 75 | (72-78) | 94 | (93-95) |
| Rectal | 93 | (92-94) | 73 | (69-77) | 95 | (94-96) | 67 | (63-71) | 90 | (89-91) |

**Appendix D**

Validation measures and 95% confidence intervals for colorectal surgery in combined physician billing and hospital inpatient data compared to the Alberta Cancer Registry overall and by year of diagnosis, stage and tumor site.

|  | **Combined Data** | | | | | | | | | |
| --- | --- | --- | --- | --- | --- | --- | --- | --- | --- | --- |
| **Factors** | **Sensitivity** | | **Specificity** | | **PPV** | | **NPV** | | **Observed Agreement** | |
|  | % | (95% CI) | % | (95% CI) | % | (95% CI) | % | (95% CI) | % | (95% CI) |
| **All** | 97 | (96-97) | 68 | (65-71) | 95 | (94-95) | 79 | (77-82) | 93 | (92-94) |
| **Year** |  |  |  |  |  |  |  |  |  |  |
| 2000 | 97 | (96-98) | 64 | (56-71) | 95 | (93-96) | 79 | (72-86) | 93 | (92-94) |
| 2001 | 97 | (95-98) | 62 | (55-69) | 93 | (92-95) | 77 | (70-83) | 91 | (89-93) |
| 2002 | 97 | (96-98) | 65 | (58-72) | 95 | (93-96) | 78 | (70-84) | 92 | (91-93) |
| 2003 | 97 | (96-98) | 74 | (67-80) | 96 | (94-97) | 81 | (75-87) | 94 | (93-95) |
| 2004 | 97 | (96-98) | 72 | (66-78) | 94 | (93-96) | 84 | (78-89) | 93 | (92-94) |
| 2005 | 96 | (95-97) | 69 | (62-75) | 94 | (93-95) | 77 | (71-83) | 92 | (91-93) |
| **Stage** |  |  |  |  |  |  |  |  |  |  |
| I | 91 | (90-93) | 88 | (47-100) | 100 | (99-100) | 5 | (2-11) | 91 | (89-93) |
| II | 99 | (99-100) | 41 | (26-58) | 99 | (98-99) | 52 | (34-69) | 98 | (97-99) |
| III | 99 | (99-100) | 70 | (50-86) | 100 | (99-100) | 59 | (41-76) | 99 | (99-99) |
| IV | 99 | (98-99) | 64 | (61-67) | 80 | (78-82) | 98 | (96-99) | 85 | (83-87) |
| Missing | 88 | (85-91) | 78 | (74-82) | 84 | (80-87) | 84 | (80-88) | 84 | (82-86) |
| **Tumor Site** |  |  |  |  |  |  |  |  |  |  |
| Colon | 97 | (96-97) | 73 | (69-76) | 95 | (95-96) | 80 | (77-83) | 93 | (92-94) |
| Rectal | 97 | (96-97) | 60 | (56-65) | 93 | (92-94) | 78 | (74-82) | 91 | (90-92) |
